# Estimation of Temporal Gait Parameters Using a Wearable Microphone-Sensor-Based System

**DOI:** 10.3390/s16122167

**Published:** 2016-12-17

**Authors:** Cheng Wang, Xiangdong Wang, Zhou Long, Jing Yuan, Yueliang Qian, Jintao Li

**Affiliations:** 1 Pervasive Computing Research Center, Institute of Computing Technology (ICT), Chinese Academy of Sciences (CAS), Beijing 100190, China; xdwang@ict.ac.cn (X.W.); longzhou@ict.ac.cn (Z.L.); yuanjing@ict.ac.cn (J.Y.); ylqian@ict.ac.cn (Y.Q.); 2 University of Chinese Academy of Sciences, Beijing 100049, China; 3 Beijing Key Laboratory of Mobile Computing and Pervasive Devices, Beijing 100190, China; jtli@ict.ac.cn

**Keywords:** gait analysis, temporal parameter estimation, footstep sound, microphone sensor, wearable device

## Abstract

Most existing wearable gait analysis methods focus on the analysis of data obtained from inertial sensors. This paper proposes a novel, low-cost, wireless and wearable gait analysis system which uses microphone sensors to collect footstep sound signals during walking. This is the first time a microphone sensor is used as a wearable gait analysis device as far as we know. Based on this system, a gait analysis algorithm for estimating the temporal parameters of gait is presented. The algorithm fully uses the fusion of two feet footstep sound signals and includes three stages: footstep detection, heel-strike event and toe-on event detection, and calculation of gait temporal parameters. Experimental results show that with a total of 240 data sequences and 1732 steps collected using three different gait data collection strategies from 15 healthy subjects, the proposed system achieves an average 0.955 F1-measure for footstep detection, an average 94.52% accuracy rate for heel-strike detection and 94.25% accuracy rate for toe-on detection. Using these detection results, nine temporal related gait parameters are calculated and these parameters are consistent with their corresponding normal gait temporal parameters and labeled data calculation results. The results verify the effectiveness of our proposed system and algorithm for temporal gait parameter estimation.

## 1. Introduction

Gait analysis (GA) is the systematic research of human walking locomotion, and it has been widely used in health diagnostics [1] or rehabilitation [2] for tasks such as assessing balance and mobility in abnormal gait patients before treatment and monitoring recovery status after treatment. Objective and quantitative measurements of gait parameters are necessary for better management of rehabilitation. Measurements of gait temporal parameters are used for the evaluation of lower extremity disorders and for the quantification of their subsequent improvement after treatment [3]. Various instruments and methods have been developed to assist in the study of gait analysis. Most big hospital and rehabilitation centers use multi-camera motion capture systems and force platforms to measure quantitative and accurate gait spatial-temporal parameters [4,5], but this approach has some disadvantages such as expensive cost, a large footprint, and specialized setups, etc. Thus it cannot meet the requirement of daily monitoring, especially for abnormal gait patients (stroke, Parkinson, multiple sclerosis, etc.) at home after leaving the hospital or rehabilitation center. Therefore, quantitative, low-cost, portable and wearable GA approaches and equipment are attracting more attention and becoming popular [6]. Foot switches or pressure sensors (insole) are used for temporal parameter estimation [7,8,9,10]. These techniques generally provide unsatisfactory results for abnormal walking and difficult sensor attachment [3]. Inertial sensors, which can be embedded in wearable and portable electronic devices, have been widely used in gait recognition for individual identification [11,12], gait action recognition [13] and gait analysis [14,15,16,17] because they are inexpensive, not limited by environmental conditions, low-power and tiny. Most wearable GA methods only use inertial sensor-based methods [14,15,16,17]. The temporal parameters of inertial sensor-based methods are mainly measured by threshold-based peak detection methods, so generally it is not easy to achieve high detection accuracy because it’s almost impossible to find a fixed threshold adaptable to many kinds of conditions. In addition, as we know that inertial sensors have chipset drift problems and integral computation accumulated errors [17], thus requiring a lot of work and processing to reduce the noises in the signals recorded by them.

On the other hand, it has been proved that footstep sounds can provide useful and important information during walking [18,19], but the microphones which were used in most previous studies were not wearable. Although a microphone-based wearable prototype device was demonstrated by a Korean company [20], it was implemented to determine the walking quality by single foot use, and was not used for GA parameter measurements. Microphone sensors are useful for wearable GA because they are tiny, low-cost, portable, etc., and a microphone sensor can get gait information directly from footstep sounds generated by the impact between someone’s foot and the floor as he or she moves around, so it can assist in the study of temporal parameter measurements. Besides these advantages, microphone sensors can more directly represent the foot-friction-floor problems caused by insufficient foot lift in some abnormal gait patients. In general, microphone sensors can be a promising technology for wearable GA studies.

The main contribution in this paper is the presentation of a study mode using wearable microphone sensors for the study of gait analysis fields. We first present a novel, low-cost, wireless, wearable and microphone-sensor-based system to collect footstep sound signals during walking, and to our knowledge this is the first time microphone sensors have been used in wearable gait analysis. Then, based on this system, a gait analysis algorithm is proposed for estimating the temporal parameters of gait. The algorithm fully uses signal fusion of two feet footstep sounds and includes three stages: footstep detection, heel-strike event and toe-on event detection, and gait temporal parameter calculation. Finally, experiments are conducted to assess the effectiveness of our proposed system and algorithm.

## 2. System Overview

**Figure 1:**
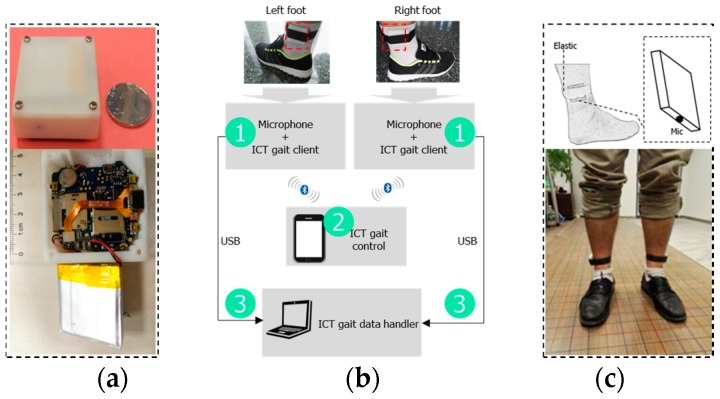
(**a**) Prototype device; (**b**) Software (SW) + hardware (HW) block diagram of the system (green numbers indicate the running sequence of system); (**c**) Wearing method.

The system firstly collects footstep sound data, which includes hardware (HW), software (SW) and data collection procedures. After the data collection, it should then be analyzed by the data analysis module. The details of our system are shown in Figure 1.

### 2.1. Hardware

We present an ankle-worn prototype device/board (our reference design is the Podoor PW308s [21]) to collect footstep sound data from both feet as shown in Figure 1a. A microphone is the data collection sensor. The prototype specification list is given in Table 1.

In actual use for this work, to protect the HW board from external impacts and make it comfortable to wear on the ankles, it was cased into an acrylic cuboid (4.3 cm × 4.8 cm × 1.5 cm) by a 3D printer and an elastic band is used for attaching it to the ankle tightly. In addition, to reduce the acoustic noise from the environment, the microphone is covered with a layer of sponge foam. In the remainder of this paper, we call the HW prototype device the test node.

### 2.2. Software

The system related software and programs are listed in Table 2.

The test node preinstalls the Google Android operating system, so it’s more easy and convenient for us to develop SW on it. We developed an Android APK program (we call it the ICT gait client) to simultaneously read microphone sensor data from the test nodes worn on both feet. Our ICT gait client program captures the footstep sound data of both feet sampled at 8 kHz in PCM format, mono channel and 16-bit resolution through the microphone while the user is walking. After installing the ICT gait client program and turning both test nodes on, the program runs automatically as an Android service. In addition, one control terminal (we use an Android smartphone) is needed to connect and transmit control commands to the two test nodes via Bluetooth, and the ICT gait control program should be installed on it (Figure 1b).

### 2.3. Data Collection Procedure

As it is shown in Figure 1b, the data collection procedure can be divided into the following stages:

(1) Two test nodes are attached to the lateral side of a test person’s two ankles with a tight elastic band, with the test nodes’ microphone holes facing the ground (Figure 1c). Both test nodes power on and the ICT gait client program is running automatically, but sensor data capture has not begun (green sequence number 1 in Figure 1b).
(2) The ICT gait control program connects to the ICT gait client program of the two feet via Bluetooth (green sequence number 2 in Figure 1b).
(3) When the test person starts walking, the ICT gait control program sends a sensor data collection start command to both ICT gait client programs, thus the two test nodes begin to record the footstep sound data synchronously.
(4) When the person stops walking, the ICT gait control program sends sensor data collection stop commands to both ICT gait client programs. Thus all gait data are recorded automatically by the two test nodes, respectively, by the ICT gait client programs.

Finally, for further data analysis, the data recorded by the two test nodes will be copied to the ICT gait data handler through a USB connection (green sequence number 3 in Figure 1b).

### 2.4. Data Analysis

After gait data collection, the ICT gait data handler in Table 2 is the gait data analysis program which mainly includes the gait parameter estimation algorithm (in the next section, we provide more details about the estimation algorithm). At present we implement gait analysis at the PC side. This means that after data capture, all the data should be manually copied to a PC through a USB connection for analysis. In a next step in our work we will consider deploying and implementing our gait data analysis in the cloud.

## 3. The Gait Temporal Parameter Estimation Algorithm

As a part of our gait data analysis module, in this section, a gait analysis algorithm is proposed for estimating the temporal parameters of gait. As we know, the basic and key process for gait analysis is to detect every footstep, namely, to detect the accurate timing of every step. After detecting every footstep, more details of the gait, such as footstep heel events and toe events can be detected accordingly. Thus more gait temporal parameters can be calculated: cadence, gait cycle time, single step time, etc. In this section we firstly describe the characteristics of normal walking footstep sounds, and then based on these characteristics, we present our algorithm for gait temporal parameter estimation.

### 3.1. The Characteristics of Footsteps

**Figure 2:**
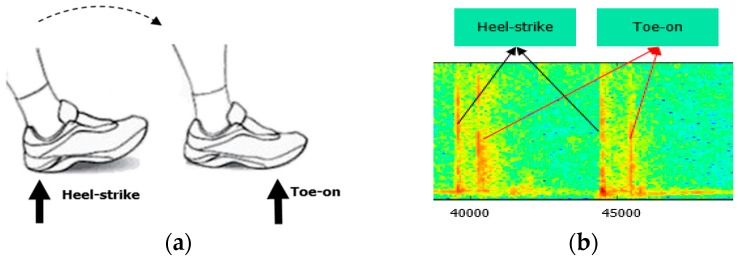
(**a**) Acoustic impacts of footstep events; (**b**) Acoustic impact signals of a footstep in a spectrogram.

As we know, a normal gait cycle includes a stance phase (60%) and a swing phase (40%) [17], and only in the stance phase, will the foot contact the ground and generate a walking sound impact. A footstep sound impact consists of several sub-impacts which are caused by different parts of one’s foot, e.g., the calcaneus touching the ground, metatarso-phalangeal ground touching and the phalanges touching the ground, etc. [18]. The different sub-impact forms indicate different human moving styles, e.g., walking, running, etc. In this paper, we only discuss walking. Usually normal walking includes two obvious sound sub-impacts: a heel-strike event and a toe-on event (Figure 2).

Here we define the heel-strike event is the calcaneus area (heel) touching the ground and the toe-on event is the metatarso-phalangeal (sole) area touching the ground. Therefore in this section, we will mainly analyze and handle these two sound event signals that happen during walking, while other related noise sounds, which also happen at the same time during walking, such as the wind noise caused by the foot swinging in the air, or friction sounds between clothes and the moving body, etc., are considered insignificantly low relative to footstep sounds and these noises are not discussed in this paper.

### 3.2. Estimation Algorithm

**Figure 3:**
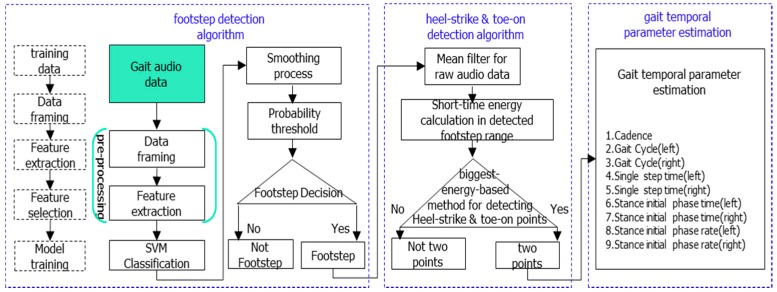
Flowchart of the temporal gait parameter estimation algorithm.

The flowchart of the gait temporal parameter estimation algorithm is shown in Figure 3. The algorithm includes three stages: footstep detection, heel-strike event and toe-on event detection, and gait temporal parameter calculation. In the following subsections, we will explain our estimation algorithm step by step in the order it’s shown in Figure 3.

#### 3.2.1. Footstep Detection Algorithm

##### Model Training

We use a classification model to detect footstep from consecutive raw audio data obtained from the system, so firstly model training is needed. All the training audio data are computed every 80 samples (0.01 s at the sample rate 8 kHz), while a frame size of 200 samples is applied (every frame is 0.025 s at the sample rate 8 kHz). Normally one touch event (from heel touching the ground to toe touching the ground) takes about 0.1 s, and this data segment method guarantees that a sufficient number of frames are included within one normal touch event. Some of the most frequently used audio features in speech processing are extracted and in total 36 features are selected for our classification model after a frame-level classification performance experiment where the accuracy was about 95% using 5-fold cross validation. The 36 features are:

- Correlation coefficient
- Sub-band energy (0–4 kHz) (the number of sub-bands is 10 by average division)
- Zero crossing rate
- LPCC (12 dimension)
- MFCC (12 dimension)

**Figure 4:**
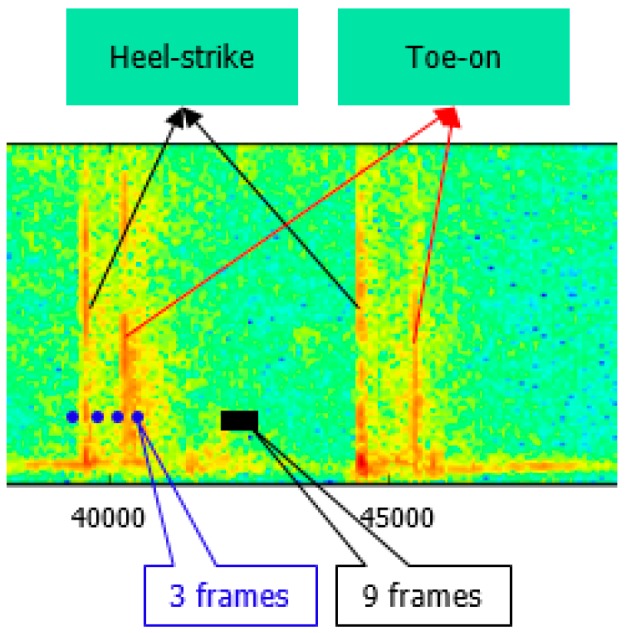
Spectrogram of footstep and training sample selection (blue ones are positive samples, in total 3 × 4 = 12 frames for each step; black ones are negative samples, in total nine frames for each step).

SVM is selected as the classification model (SVM-related parameters are as follows: kernel is the radial basis function, C = 2048, gamma = 0.5). Before model training, we need to select the positive samples and negative samples. Considering the typical characteristics of walking footsteps where heel-strike and toe-on are obvious events for generating footstep audio data, one reasonable sample selection policy is: three frames at the two sides of the touch point of heel and toe, respectively, as positive samples, and nine frames at the middle of two neighboring steps as negative samples (Figure 4).

##### Footstep Detection

After the pre-processing of audio gait data, it should be put into the SVM classification which was obtained after training (Figure 3). According to the training method mentioned above, the SVM classification is a frame-level probability model. This means that after SVM classification, it can determine whether an audio frame corresponds to a footstep or not. In fact, our objective is to detect which phase of the audio is a footstep in the consecutive walking audio data sequences. Before detecting footsteps in audio data sequences, a smoothing process is needed. Here a low-pass-filter (LPF) is designed (cut-off frequency is 0.1 Hz, number of coefficients is 101) for smoothing the probability results after SVM:

(1) $$y\left(n\right)=\overset{100}{\underset{i=0}{\sum }}\left(w_{i}x\left(n\right)\right)$$

where x(n) is the input signal after classification model (SVM) calculation and $w_{i}$ are the LPF coefficients.

**Figure 5:**
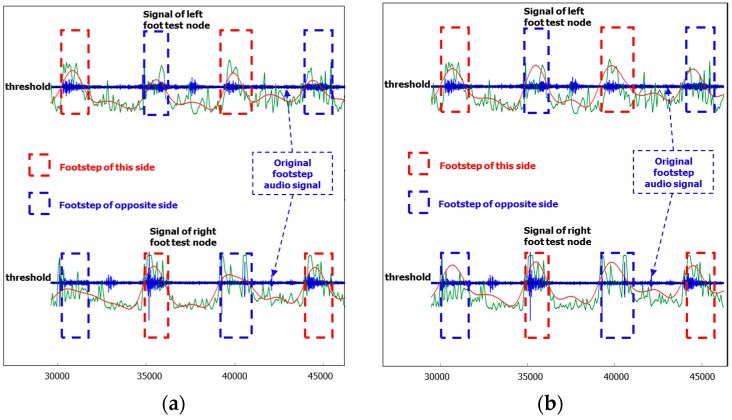
(**a**) Low-Pass-Filter smoothing result (red) for the probability curve (green); (**b**) Footstep detection by two-footstep-audio signal fusion.

Figure 5a shows the result where the original footstep audio is indicated by a blue curve, the probability curve after SVM is indicated by a green curve, and the red curve is the smoothing result after LPF. It shows that there is an obvious peak (red curve) for each footstep, so we can detect these peaks by a probability threshold value. If a peak is higher than the threshold value, it can be judged as a footstep; otherwise, it’s not. At the same time, other information that can be seen in this figure is that when a footstep happens both test nodes (left and right) can capture the audio of this footstep, but the opposite side footstep’s peak is a little flatter than the current side footstep’s peak because the opposite side footstep’s audio signal is relatively a little smaller than the actual side footstep’s audio signal. This is due to the fact that the opposite side’s foot is farther away from the microphone than this side’s foot. To a certain extent, this newly found information also proves that our smoothing process for footstep detection is correct. However, according to this newly found information, we also find that there will be missing footstep detection signal errors with only single foot signals. For example, as shown in Figure 5a, the first red curve peak is detected as a footstep by the left foot signal, but it’s not a footstep according to the right foot signal, so usually footstep detection by a single foot’s audio signal cannot get good results.

Based on the above discussion, we propose a footstep detection algorithm which fully uses two-footstep-audio signal fusion: (1) during the training phase, footstep audio data of both feet will be added into a training set; (2) for detection of footsteps, two-footstep-audio signals will be used to judge whether the signal is a footstep or not. One reasonable solution is that the sum of the two (left and right) footstep-audio probabilities, which should be smoothly processed by LPF in advance, can be used as a judging condition. Here we set the threshold value as 0.8 instead of 1.0 because at the same moment the signal obtained from the opposite side footstep’s audio signal is relatively a little smaller than the current side footstep’s audio signal:

(2) $$\left\{ \begin{array}{c} {P_{\mathrm{left}}}^{\left(i\right)}+{P_{\mathrm{right}}}^{\left(i\right)}\geq 0.8,\;\mathrm{it}’s\;a\;\mathrm{footstep}\\ {P_{\mathrm{left}}}^{\left(i\right)}+{P_{\mathrm{right}}}^{\left(i\right)}<0.8,\;\mathrm{it}’s\;\mathrm{not}\;a\;\mathrm{footsetp} \end{array}\right.$$

where ${P_{\mathrm{left}}}^{\left(i\right)}$ and ${P_{\mathrm{right}}}^{\left(i\right)}$ is the footstep probability of the left foot and right foot’s sound signal at position (i). However, for light footsteps (e.g., a light weight person wearing shoes with very soft soles), this solution sometimes can’t get good results because the footstep-audio probability is almost zero. Thus the sum of the two (left and right) footstep-audio probabilities will be less than the threshold value, and if the threshold value is reduced more, then the detection errors will increase. Another problem of this solution is that after a footstep is confirmed, it’s hard to recognize whether it corresponds to a left footstep or a right footstep.

To address this problem, another solution is that after LPR smoothing, the bigger probability of two footstep-audio signals (the signals were generated by one footstep, but captured by both feet’s test nodes at the same time) can be used as the decision criterion:

(3) $$\left\{ \begin{array}{c} P^{\left(i\right)}={P_{\mathrm{left}}}^{\left(i\right)},\;\mathrm{if}\;{P_{\mathrm{left}}}^{\left(i\right)}\geq {P_{\mathrm{right}}}^{\left(i\right)},\;\mathrm{it}’s\;a\;\mathrm{left}\;\mathrm{foot}\;\mathrm{footstep}\\ P^{\left(i\right)}={P_{\mathrm{right}}}^{\left(i\right)},\;\mathrm{if}\;{P_{\mathrm{left}}}^{\left(i\right)}<{P_{\mathrm{right}}}^{\left(i\right)},\;\mathrm{it}’s\;a\;\mathrm{right}\;\mathrm{foot}\;\mathrm{footstep} \end{array}\right.$$

The essence of this solution is that at the same moment the signal obtained from this side footstep’s audio signal is relatively bigger than the audio signal from the opposite side footstep (Figure 5a), so this side’s footstep signal is used for the footstep detection.

By this detection algorithm, we can get the smoothing result curve shown in Figure 5b with the same audio signal of Figure 5a. From the result (shown by a red line), we can clearly and exactly find the footsteps.

#### 3.2.2. Heel-Strike & Toe-on Detection Algorithm

After footstep detection, we know which audio signal range includes the footsteps, and this range is closely related to or includes the footstep’s heel and toe event. However, our objective is to find out the time point of heel-strike and toe-on in this detected footstep range. Before detecting these two events, a mean filter is implemented for smoothing of raw audio signal. In our work, the filter size used is 5 frames.

**Figure 6:**
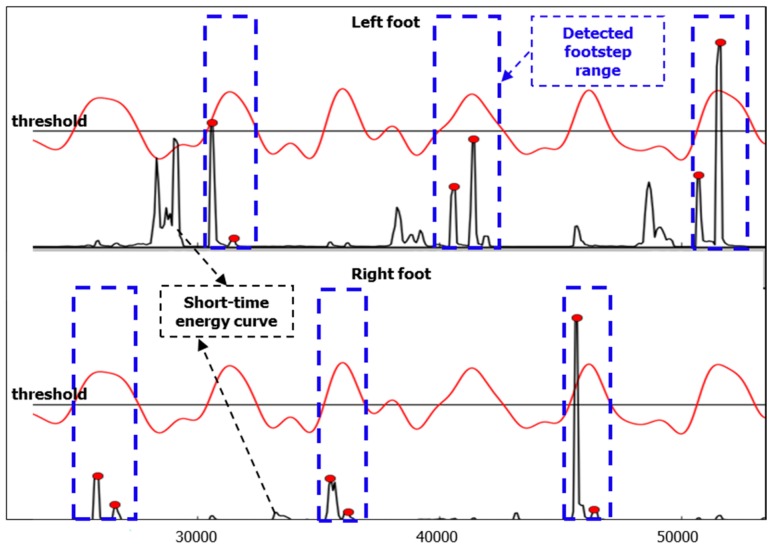
Low-pass-filter smoothing result (red curve), short-time energy (black curve), detected footstep (blue dotted line rectangle), detected events of heel-strike and toe-on (red circular dots).

Since the footstep sound is an impact sound between the shoe and the floor, in a footstep there is a relatively high signal-noise ratio. In this case, short-time energy is a good choice for detecting the heel-strike and toe-on event from the detected footstep range. The short-time energy of *l* position is calculated by a Fourier transform:

(4) $$X\left(n\right)=\overset{N-1}{\underset{k=0}{\sum }}x_{k}e^{i\frac{2\pi }{N}kn}$$

(5) $$E\left(l\right)=\overset{high\;freq}{\underset{n=low\;freq}{\sum }}{\left(X\left(n\right).real\right)}^{2}+{\left(X\left(n\right).imag\right)}^{2}$$

where *l* = 0, …, *L*, *L* is the audio signal length, $x_{k}$ is the input signal and *N* is the frame size. As shown in Figure 6, the detected footstep range is represented by a blue dotted line rectangle, and short-time energy curve is indicated using the black curve. The beginning point of each detected footstep range is the first cross point of the red low-pass-filter curve and the black threshold line, and the end point of each detected footstep range is the second cross point of the red low-pass-filter curve and the black threshold line.

**Figure 7:**
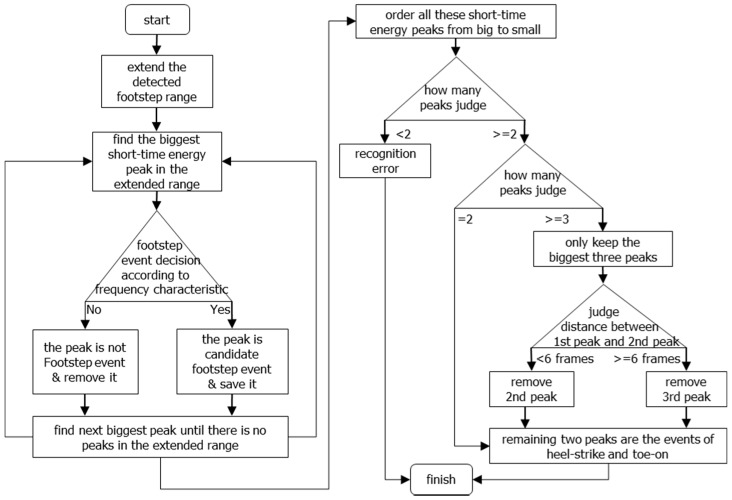
Diagram of biggest-energy-based method for detection of heel-strikes and toe-ons.

In fact, from the short-time energy curve we can see that there are still some noises aside from the heel-strike and toe-on sound events within the detected footstep range. Thus, after calculating the short-time energy, we use a biggest-energy-based method for finally detecting heel-strike points and toe-on points within the detected footstep range. The diagram of the method is shown in Figure 7, and the detailed flow is as follows:

(1) Because our footstep detection algorithm proposed in the last section is based on a threshold method, there is the possibility that some heel-strike points and toe-on points may be missed, so to reduce this risk, we extend the footstep detection range. In our work, we extend the range by 10 frames: five frames for the left border and five frames for the right border;
(2) Select the biggest short-time energy peak in the extended range;
(3) As we know, footstep impact sound signals range from low frequency to high frequency in the frequency spectrum, but non-footstep-sound (noise) is only low frequency (Figure 4), so according to this information, we can determine whether the selected biggest peak is a footstep-sound or not. If it is not a footstep sound, then we can remove it and find out the next biggest short-time energy peak in the same range. If it is footstep sound, then we retain it and find the next biggest one. Finally in each detected footstep range, all the short-time energy peaks which are heel-strike point and toe-on point candidates are found.
(4) Order all these short-time energy peaks from big to small, and only keep the biggest three if there are more than three peaks; if there are only two peaks left, then this processing is finished; if there is only one peak or no peak, then it is a detection error, and this processing run is finished.
(5) With three peaks, judge whether the distance between the 1st one and 2nd one is very small. If it’s very small (in our work, the distance judging condition is six frames), it means that the distance is smaller than a normal footstep’s distance between heel-strike and toe-on, then remove the 2nd one and keep 1st one and 3rd one. If it’s not very small, then remove the 3rd one, and keep 1st one and 2nd one.
(6) Finally, the remaining two peaks are the heel-strike point and toe-on point, respectively according to the chronological order. This means that the 1st one peak is a heel-strike point, and the 2nd peak a toe-on point. They are shown with red circular dots in Figure 6, and within one footstep, the left red dot is a heel-strike point, and the right red dot is a toe-on point, respectively.

Therefore, we can get the two points from the detected footstep sound range: the heel-strike point and toe-on point.

#### 3.2.3. Gait Temporal Parameters Estimation

According to the abovementioned methods, we find out the heel-strike point and toe-on point from each detected footstep. Then in this paper, nine related temporal parameters, which are important for assessing the regularity, symmetry and mobility of gait, were estimated [3,22]:

##### Cadence (Steps/Min)

This is step number per minute. Let *N* be the number of steps taken over the time period *t* (in second). Cadence can thus be expressed as:

(6) $$C=60\frac{N}{t}$$

Normally a human’s cadence is 95–125 steps/min.

##### Gait Cycle (s)

This is the time period during walking from when one foot heel contacts the ground to when that same foot heel contacts the ground again, so a gait cycle is divided into the left foot’s cycle and the right foot’s cycle ($GC_{left},GC_{right}$). Each foot’s gait cycle consists of two phases, namely the stance phase and the swing phase. The stance phase can be further divided into load phase, foot-flat phase and push phase [23]. In this work, we call the load phase the stance initial phase.

##### Single Step Time (s)

This is the time period during walking in which one foot heel contacts the ground to when the other foot heel contacts the ground, so a single step time is divided into left foot’s and right foot’s ($SST_{left},SST_{right}$).

##### Stance Initial Phase Time (s)

This is the time period during walking from when one foot heel contacts the ground to when that same foot toe contacts the ground:

(7) SIPT=toeon−heelstrike

Stance initial phase time is divided into left foot’s and right foot’s ($SIPT_{left},SIPT_{right}$).

##### Stance Initial Phase Rate (%)

This corresponds to the proportion of stance initial phase in a whole gait cycle. It is defined by the following formula:

(8) $$SIPR=\frac{SIPT}{GC}\times 100\%$$

Stance initial phase rate is also divided into left foot’s and right foot’s ($SIPR_{left},SIPR_{right}$).

Regarding to the calculation method of these nine parameters which is mentioned above, it should be pointed out that except for cadence, the calculation method of gait cycle is for one cycle, and the other six parameters are measured for one footstep. The final calculation method of each parameter for one subject is to take an average value.

## 4. Experiments

To show the effectiveness of our proposed system and algorithms, we conducted some experiments. The experiments include two parts: experimental data and experimental results.

### 4.1. Experimental Data

We establish an ICT_Gait database containing two feet audio data for each participating subject. Meanwhile, the corresponding temporal data in the database are labeled with the ground-truth.

#### 4.1.1. Data Collection Environment

**Figure 8:**
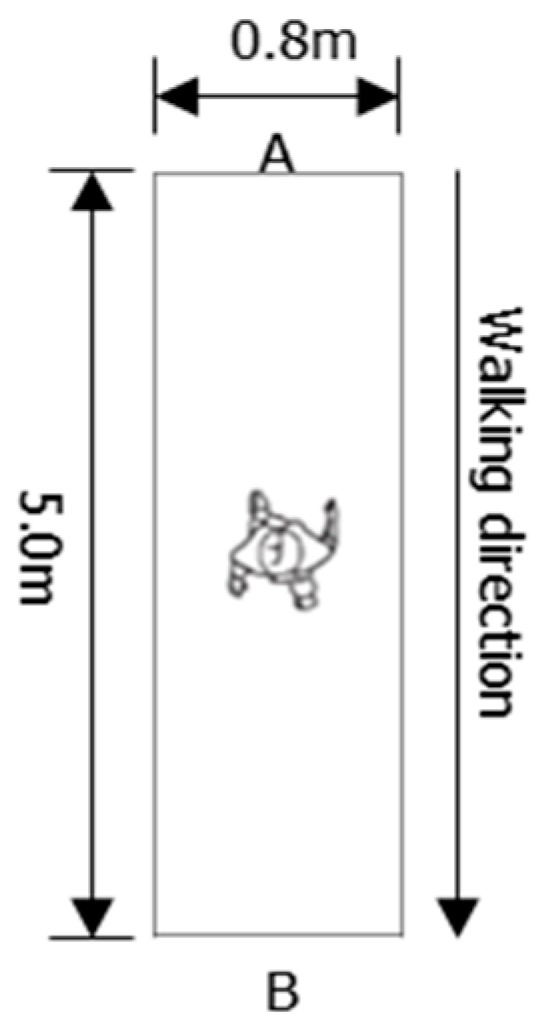
Data collection environment (top down view).

For data collection, we set up a test lab at our research center, a room with the following dimensions: height 2.85 m, width 3.85 m and length 6.45 m. In this room there is a rectangle test ground of 5 × 0.8 square meters, as shown in Figure 8.

#### 4.1.2. Participating Subjects

In our work, 15 healthy subjects volunteered to participate in our research. Before the tests, all the subjects were requested to sign the test agreement document and each one received a small gift of roughly 8 USD in value. The details for the total 15 participating subjects are: nine (59%) were males and six (41%) females. The ages of the subjects ranged from 21 to 54 years old, with an average of 33.13 years old. Their heights ranged from 155 cm to 189 cm, with an average of 168.07 cm. As for the weight of the subjects, it ranged from 48 kg to 98 kg, with an average of 64.67 kg.

#### 4.1.3. Data Collection Strategy

Participating subjects were asked to walk straight at a normal speed from the left (marked with A) to the right (marked with B), then turn around and come back. Considering that footstep sounds have a big relationship with the ground and shoe type, we added the following two variations: (1) shoe type: sneakers (soft sole), leather shoe (hard sole) and (2) ground type: cement, wood.

In addition, we add another variation that asks the subjects to carry a 5 kg weight bag on the back when walking to confirm whether overloading has any effect on their gait. In all, there are in total three different data collection variables (shoe type, ground type, and 5 kg bag load) for each subject, and each variant has two selections. Combined with the walking direction (walking from A to B generates one data sequence, and walking from B to A generates another data sequence), we can get in total 16 data sequences for each subject, so the whole database has 240 data sequences (15 × 16).

#### 4.1.4. Data Collection Method

Each participating subject walks back and forth in the 5 m × 0.8 m rectangle test ground with three different data collection strategies, and the method is same as the one described in Section 2.3.

#### 4.1.5. Data Labeling Method and Content

For the labeling of gait temporal parameters, in this work we present a simple method to accurately label data after walking. We directly use the audio data recorded by two foot-worn test nodes. In order to guarantee the accuracy of the labeled data, our labelling process is divided into two steps:

Firstly, experienced audio experts of our lab listen to each footstep’s audio data, and manually record the time position of each footstep event (heel and toe touch ground).

**Figure 9:**
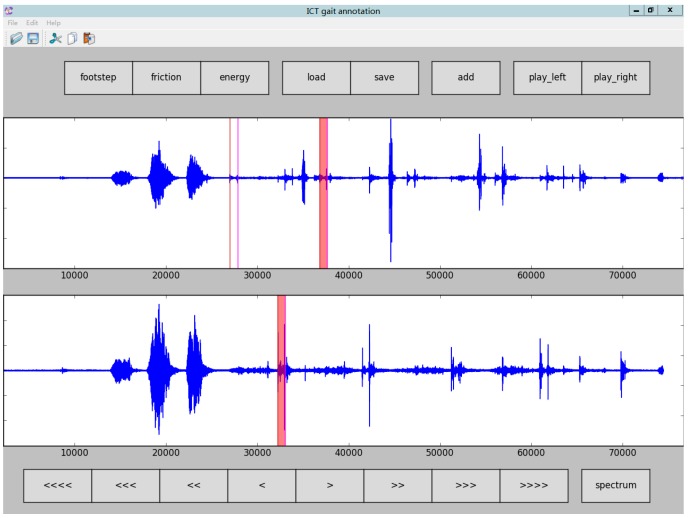
Screenshot of the temporal parameter label tool.

Secondly, a temporal parameter label tool (ICT gait annotation) was developed by us to correct and revise the first step’s mark result. A screenshot of this tool is shown in Figure 9. Normally it is not easy if we only depend only on the audio signal amplitude information determined by experienced audio experts, so the energy curve, frequency spectrum, and the bandwidths are computed and plotted on the screen to assist the labeling process.

According to this method, the label content is the time of heel-strike and toe-on, and the time is represented as the audio sample number.

#### 4.1.6. Experimental Data

Considering the stability of each gait data sequence, before the experiment, the first and last footstep of each data sequence are removed, and our database is divided into a training set, validation set and the test set for the experiments (Table 3).

### 4.2. Experimental Results

To estimate gait temporal parameters, footstep detection is the first step. After footstep detection, then heel-strike and toe-on can be detected accordingly. Only if both heel-strike and toe-on points are detected, can the nine gait temporal parameters (cadence, gait cycle (left, right), single step time (left, right), stance initial phase time (left, right), stance initial phase rate (left, right)) be calculated. In the follow subsections, we give the experimental results after running our algorithms based on the data shown in Table 3.

#### 4.2.1. Footstep Detection Performance

**Figure 10:**
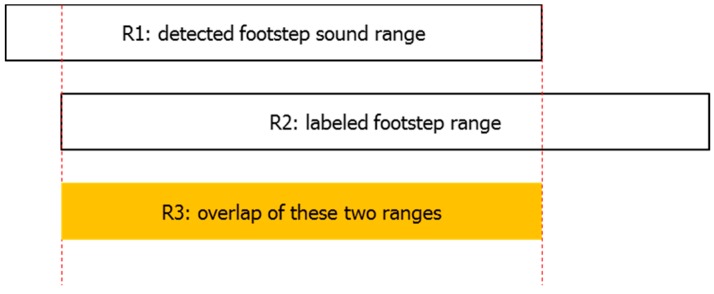
Relationship between detected footstep sound range and labeled footstep range.

According to our proposed footstep detection algorithm, we can get a footstep sound range. Our experiment is designed to compare detected footstep sound ranges with labeled footstep ranges. Here we define an overlap-threshold-rate value which is the ratio of overlap of these two ranges to the minimum of these two ranges as shown in Equation (9), and the relationship between these related footstep ranges is shown in Figure 10.

(9) $$\mathrm{Overlap}-\mathrm{threshold}-\mathrm{rate}=\frac{R3}{minimum\left(R1,R2\right)}\times 100\%$$

**Figure 11:**
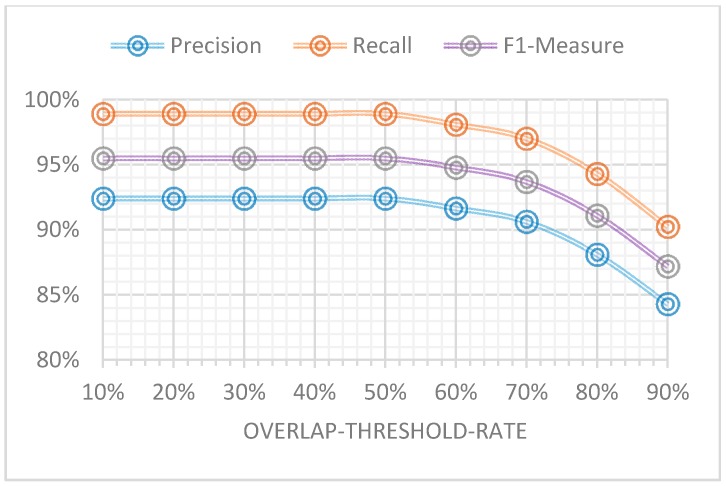
Relationship between footstep detection performance and overlap-threshold-rate.

We did experiments to determine the relationship between overlap-threshold-rate value and footstep detection precision rate, recall rate and F1-measure. The results are shown in Figure 11. From it we can see that as the overlap-threshold-rate increases, the footstep detection performance is declining, and as overlap-threshold-rate decreases, the footstep detection performance is improving. However, if the overlap-threshold-rate goes below 50%, the performance remains the same. This proves that the detected footstep sound ranges and the labeled footstep ranges have good consistence and as a whole there is about 50% overlap between these two ranges. Considering that our objective is to calculate the gait temporal parameters and footstep detection is the first step in accomplishing our objective, in theory the higher the footstep detection performance gets, the better the calculation result of gait temporal parameters will be. This is because there are less missing footsteps. Consequently, in this paper we use 50% as the overlap-threshold-rate value because of its higher footstep detection performance.

Therefore, in the footstep detection experiments, if the real overlap rate is more than the overlap-threshold-rate value, then it’s matched; otherwise, it’s not matched. The results are shown in Table 4.

From them we can see that the average high recall rate (98.92%) and high F1-measure (0.955) prove that footstep sounds can directly provide the gait information and microphones are beneficial for wearable sensor gait analysis. This result also can be proved from Figure 11, where even if the overlap-threshold-rate value equals 90%, the F1-measure of footstep detection is also more than 0.85 (85%). From Table 4, we also see that average precision rate (92.41%) is not very high because walking audio signal may be affected more or less by some noises such as the wind noise caused by the foot swinging in the air, friction sounds between clothes and the subject’s moving body and other environmental noises during forward walking. These noises cause some detection errors (Figure 4 shows some noises occurring during normal walking). In addition, regarding the test result for the different data collection strategies (variations), the F1-measure shows that the performance is almost same for each variant. This is because we used all the variation data for training, so our model will adapt to different variations. On the other hand, this result also proves that our proposed footstep detection algorithm has good robustness.

#### 4.2.2. Heel-Strike and Toe-on Detection Performance

**Figure 12:**
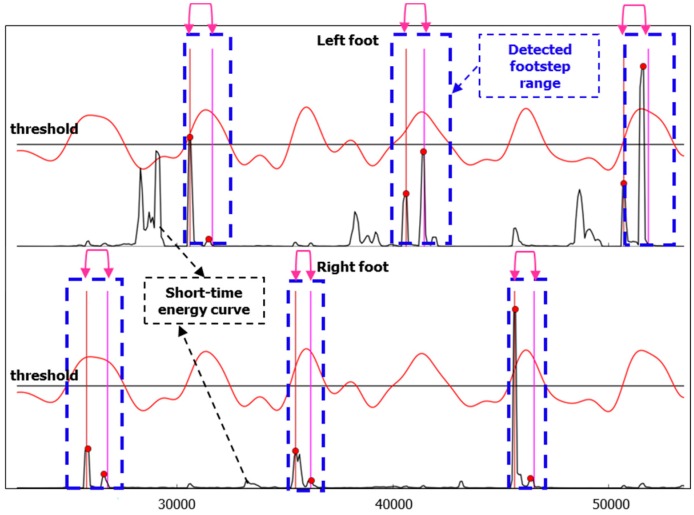
Footstep probability curve after LPF (red); short-time energy curve (black); detected footstep (blue dotted line rectangle); labeled events of heel-strike and toe-on (pink lines); detected heel-strike (left-first red dot); detected toe-on (right-second red dot).

As shown in Figure 12, for each footstep, the detected heel-strike is indicated by a left red dot and detected toe-on is indicated by a right red dot. The labeled point of heel-strike and toe-on of each footstep are described by the left and right pink lines, respectively. In our work, we set three frames for tolerance error, namely, if the deviation’s absolute value of the detected heel-strike time point from its true value which was labeled in our database is less than three frames, we determine it’s matched; if the deviation’s absolute value of the detected toe-on time point from its true value which was labeled in our database is less than three frames, we determine it’s matched.

Based on the abovementioned conditions, the experimental results of the two event detection are shown in Table 5:

From the results shown in Table 5, we can see that the method achieves an average 94.52% accuracy rate for heel-strike detection and 94.25% accuracy rate for toe-on detection. Regarding the detection of heel-strikes, the accuracy rate of leather shoes (96.77%), wood ground (96.74%), 5 Kg-load-yes (94.57%) is a bit bigger than the accuracy rate of sneaker shoes (92.18%), cement ground (92.27%), 5 Kg-load-no (94.48%). Similarly, for the detection of toe-on, the accuracy rate of leather shoes (98.39%), wood ground (96.74%), 5 Kg-load-yes (94.57%) is a bit bigger than the accuracy rate of sneaker shoes (89.94%), cement ground (91.71%), 5 Kg-load-no (93.92%). This is because for normal walking the footstep sound signals of leather shoes, wood ground and 5 Kg-load-yes is a little bigger or clearer than the footstep sound signals of sneaker shoes, cement ground and 5 Kg-load-no.

#### 4.2.3. Gait Temporal Parameter Estimation

Gait temporal parameters are calculated based on the detection results of heel-strike and toe-on. The results are shown in Table 6. From the results, we can see that for each subject the left foot parameters are almost as same as the right foot parameters, which shows the good symmetry between left root and right root because all the test subjects are healthy. We also can see the average cadence is 104.15 steps/min, average gait cycle is left 1.164 s and right 1.157 s, average single step time is left 0.574 s and right 0.585 s, average stance initial phase time is left 0.095 s and right 0.100 s, and average stance initial phase rate is left 8.20% and right 8.64% for whole gait cycle, which is consistent with the normal gait temporal parameters presented in [24]. This proves the effectiveness of our system and estimation algorithms.

We also compared our temporal parameter estimation results with labeled data calculation results (Table 7). From these two tables, we can see the estimation result of average cadence (104.15 steps/min), average gait cycle (left 1.164 s/right 1.157 s), average single step time (left 0.574 s/right 0.585 s), average stance initial phase time (left 0.095 s/right 0.100 s) and average stance initial phase rate (left 8.20%/right 8.64%) are almost perfectly matched with their corresponding labeled data calculated results (103.67 min/steps, left 1.161 s/right 1.160 s, left 0.579 s/right 0.583 s, left 0.111 s/right 0.113 s, left 9.52%/right 9.72%).

## 5. Discussion

The main contribution of this study is to provide a new way of using wearable microphone sensors for the study of gait analysis fields. Concretely, a novel, portable, wireless and wearable gait data analysis system and a method for gait temporally-related parameter estimation was developed and proposed in the study. The study results show that footsteps could be identified with high recall rate and high F1-measure through footstep sound signals in normal walking gait, and two important gait cycle events (heel-strike and toe-on) in one footstep could also be detected with high accuracy. Based on these detected results, nine gait-related temporal parameters were calculated. These calculated parameters fit well with their corresponding normal gait temporal parameters. In addition, the temporal parameters calculated by our method and those obtained from labeled data calculation results were consistent enough that our system and algorithm could have big potential for gait analysis. In general, based on the contributions of this study, researchers in the gait analysis field can have one more choice for selecting sensors when they measure gait temporal parameters.

In addition, the signals from the microphone sensors on the ankle showed some noises, and this caused the footstep detection to have not very high precision rates (although it’s more than 90%, which is still a good result). In this paper, we detected footsteps by using a classification-model-based method and this can partially reduce the noise effects. Moreover, the system which was proposed in this paper will be mainly used for flat floors, hard floors and indoor applications with low environmental noise, so this result can be accepted. If some special application scenarios need very high precision rates, another sensor (e.g., an inertial sensor) can be considered to form a multimodal-sensor-based system. In fact, this solution has been considered for our next work.

The limitation of this study is that we only used a single sensor type (microphones) to estimate gait parameters, and with only single sensor it is hard to measure and estimate more gait parameters such as foot-flat phase, push phase, spatial parameters, etc. [17,23]. Especially for spatial parameters, inertial sensors (accelerometers) can measure them by integral computation, but as we know, inertial sensors have chipset drift problems and integral computation accumulated errors, and this error is corrected by a zero velocity update. Current detection methods of zero velocity are mainly determined by temporal parameters, therefore, the fusion of these two types of sensors has big potential for gait analysis. In further study, we will consider the use of multimodal sensor fusion for the measurement and estimation of more gait parameters. Despite this limitation, our low-cost, wireless, portable and wearable system and estimation algorithm were proved to be effective and valuable to calculate temporal gait parameters.

## 6. Conclusions

In this article we have proposed a two-foot-ankle worn system which uses microphone sensors to capture human gait. To our knowledge, this is the first time that microphone sensors have been used for wearable gait analysis. Based on the system, a study presenting an algorithm was made for gait temporal parameters estimation. The algorithm fully uses the footstep sound signal fusion of both feet and includes three stages: footstep detection, heel-strike event and toe-on event detection, and gait temporal parameter calculation. Finally, our experimental results show that with 15 healthy subjects, a total of 240 data sequences and 1732 steps from three different gait data collection strategies, we can achieve an average 0.955 F1-measure for footstep detection, an average 94.52% accuracy rate for heel-strike detection and a 94.25% accuracy rate for toe-on detection. By using these detection results, nine temporally-related gait parameters are calculated and these parameters are consistent enough with their corresponding normal gait temporal parameters and labeled data calculation results. The results verify the effectiveness of our proposed system and algorithm for gait temporal parameter estimation and gait analysis. Thus it can be expected that our wearable system and gait temporal parameter estimation method will be useful in daily life for health rehabilitation monitoring of abnormal gait patients at home after leaving the hospital or rehabilitation center, physical exercise evaluation and as a guide for sports fans, elder fall forecasting, human activity detection, etc.

In future work, we will augment the experiment’s database to include more subjects such as patients with various problems (stroke, Parkinson’s disease, multiple sclerosis, etc.) for generalization of our study. We also plan to implement multimodal sensors to reduce the noise effects of footstep detection and estimation of more gait parameters.

## Figures and Tables

**Table 1:** Specification list of the device HW.

Part	Spec
Microphone	Omni-directional, electrets, condenser
Bluetooth	CSR, Bluetooth 4.0/3.0 + EDR
Battery	3.7 V, 600 mAH, Li-ion
Memory	1 GB RAM + 8 GB ROM
CPU	MT6572, 1.3 GHz, dual-Core

**Table 2:** System SW list.

SW Name	Target to Be Installed	Number
ICT gait client	Test node (left foot)	1
ICT gait client	Test node (right foot)	1
ICT gait control	Control terminal	1
ICT gait data handler	PC	1

**Table 3:** Data structure for the experiments.

Data Type	Audio Data			Total
	Training Set	Validation Set	Test Set	
Subjects	8	3	4	15
Number of data sequence	128	48	64	240
Number of footstep	736	267	369	1732

**Table 4:** Footstep detection results.

Data Collection Strategy	Correct Detections	Detection Errors	Missed Detections	Precision	Recall	F1-Measure
Sneaker shoe	179	12	3	93.72%	98.35%	0.960
Leather shoe	186	18	1	91.18%	99.47%	0.951
Wood ground	184	12	1	93.88%	99.46%	0.966
Cement ground	181	18	3	90.95%	98.37%	0.945
Load 5 Kg-Yes	184	12	1	93.88%	99.46%	0.966
Load 5 Kg-No	181	18	3	90.95%	98.37%	0.945
**Average**	**365**	**30**	**4**	**92.41%**	**98.92%**	**0.955**

**Table 5:** Heel-strike and toe-on detection result.

Data Collection Strategy	Total Footsteps	Heel-Strike			Toe-on		
		Correct	Error	Accuracy	Correct	Error	Accuracy
Sneaks shoe	179	165	14	92.18%	161	18	89.94%
Leather shoe	186	180	6	96.77%	183	3	98.39%
Wood ground	184	178	6	96.74%	178	6	96.74%
Cement ground	181	167	14	92.27%	166	15	91.71%
Load 5 Kg-Yes	184	174	10	94.57%	174	10	94.57%
Load 5 Kg-No	181	171	10	94.48%	170	11	93.92%
**Average**	**365**	**345**	**20**	**94.52%**	**344**	**21**	**94.25%**

**Table 6:** Estimation results of gait temporal parameters.

Test Subjects	Cadence (Steps/min)	Left Foot				Right Foot			
		Gait Cycle (s)	Single Step Time (s)	Stance Initial Phase Time (s)	Stance Initial Phase Rate (%)	Gait Cycle (s)	Single Step Time (s)	Stance Initial Phase Time (s)	Stance Initial Phase Rate (%)
Subject 1	98.64	1.225	0.604	0.097	7.90%	1.222	0.613	0.101	8.25%
Subject 2	101.99	1.197	0.584	0.116	9.70%	1.188	0.604	0.104	8.78%
Subject 3	103.54	1.165	0.572	0.093	8.02%	1.151	0.588	0.107	9.28%
Subject 4	112.46	1.065	0.533	0.075	7.08%	1.069	0.535	0.088	8.24%
**Average**	**104.15**	**1.164**	**0.574**	**0.095**	**8.20%**	**1.157**	**0.585**	**0.100**	**8.64%**

**Table 7:** Labeled data results of gait temporal parameters.

Test Subjects	Cadence (Steps/min)	Left Foot				Right Foot			
		Gait Cycle (s)	Single Step Time (s)	Stance Initial Phase Time (s)	Stance Initial Phase Rate (%)	Gait Cycle (s)	Single Step Time (s)	Stance Initial Phase Time (s)	Stance Initial Phase Rate (%)
Subject 1	98.37	1.225	0.613	0.113	9.22%	1.226	0.606	0.119	9.71%
Subject 2	100.21	1.194	0.598	0.127	10.66%	1.190	0.600	0.121	10.19%
Subject 3	103.62	1.159	0.576	0.104	8.99%	1.153	0.584	0.112	9.69%
Subject 4	112.48	1.063	0.529	0.098	9.19%	1.072	0.542	0.099	9.23%
**Average**	**103.67**	**1.161**	**0.579**	**0.111**	**9.52%**	**1.160**	**0.583**	**0.113**	**9.72%**

## Abbreviations

ICT	Institute of Computing Technology
GA	gait analysis
HW	hardware
SW	software
PCM	pulse code modulation
PC	personal computer
LPCC	Linear Prediction Cepstrum Coefficients
MFCC	Mel Frequency Cepstrum Coefficients
SVM	Support Vector Machine
LPF	low-pass-filter
